# Role of Protein Tyrosine Phosphatase Non-Receptor Type 7 in the Regulation of TNF-α Production in RAW 264.7 Macrophages

**DOI:** 10.1371/journal.pone.0078776

**Published:** 2013-11-12

**Authors:** Huiyun Seo, In-Seon Lee, Jae Eun Park, Sung Goo Park, Do Hee Lee, Byoung Chul Park, Sayeon Cho

**Affiliations:** 1 College of Pharmacy, Chung-Ang University, Seoul, Korea; 2 Medical Proteomics Research Center, Korea Research Institute of Bioscience and Biotechnology, Daejeon, Korea; 3 Department of Biotechnology, College of Natural Sciences, Seoul Women’s University, Seoul, Korea; Virginia Tech University, United States of America

## Abstract

Protein tyrosine phosphatases play key roles in a diverse range of cellular processes such as differentiation, cell proliferation, apoptosis, immunological signaling, and cytoskeletal function. Protein tyrosine phosphatase non-receptor type 7 (PTPN7), a member of the phosphatase family, specifically inactivates mitogen-activated protein kinases (MAPKs). Here, we report that PTPN7 acts as a regulator of pro-inflammatory TNF-α production in RAW 264.7 cells that are stimulated with lipopolysaccharide (LPS) that acts as an endotoxin and elicits strong immune responses in animals. Stimulation of RAW 264.7 cells with LPS leads to a transient decrease in the levels of PTPN7 mRNA and protein. The overexpression of PTPN7 inhibits LPS-stimulated production of TNF-α. In addition, small interfering RNA (siRNA) analysis showed that knock-down of PTPN7 in RAW 264.7 cells increased TNF-α production. PTPN7 has a negative regulatory function to extracellular signal regulated kinase 1/2 (ERK1/2) and p38 that increase LPS-induced TNF-α production in macrophages. Thus, our data presents PTPN7 as a negative regulator of TNF-α expression and the inflammatory response in macrophages.

## Introduction

Protein phosphorylation is a critical event in signal transduction, which regulates fundamental cellular processes such as differentiation, cell proliferation, apoptosis, immunological signaling, and cytoskeletal function [1]. Protein phosphorylation is regulated by the opposing actions of kinases and phosphatases, and, importantly, provides a means of regulating protein function. The regulated expression and activity of several protein tyrosine phosphatases (PTPs) in cells, in turn, control the duration and intensity of the activity of mitogen-activated protein kinase (MAPK), which determines the type of physiological response. The MAPK subfamily, including the c-Jun N-terminal kinase (JNK), extracellular signal-regulated kinases (ERK), and p38, act as key inflammatory mediators in the mammalian innate immune system response [2], [3]. In particular, the phosphorylation of MAPKs plays a critical role in the inflammatory response [4]. When stimulated with lipopolysaccharide (LPS), innate immune cells like macrophages release pro-inflammatory cytokines, such as tumor necrosis factor-alpha (TNF-α), interleukin-6 (IL-6), interleukin-12 (IL-12), monocyte chemotactic protein-1 (MCP-1), interferon-gamma (IFN-γ), and interleukin-10 (IL-10), through complex signaling mechanisms [5], [6]. The regulation of TNF-α expression is mediated by the adenosine/uridine-rich element (ARE) in the 3′- untranslated region of the TNF-α mRNA that represses TNF-α expression post-transcriptionally [7], [8]. MAPKs like p38, JNK, and ERK have been shown to target this ARE to increase TNF-α expression in response to LPS stimulation [9].

PTP-deficient mouse models have been used to identify the role of individual PTPs immune response regulation [10]. In LPS-stimulated RAW 264.7 cells, the activity of dual-specificity phosphatase 1 (DUSP1) protein increases dramatically, reaching its maximal level between 1 and 2 h, and then decreasing thereafter [11]. *Dusp1^−/−^* macrophages have elevated p38 and JNK activity but unchanged ERK activity [12], [13]. DUSP1 specifically inactivates JNK and p38 *in vitro* by dephosphorylating both phospho-Thr and phospho-Tyr residues of these kinases. Other DUSP members have also been identified as regulators of inflammation in innate immune cells [3]. We have previously shown that DUSP26, PTPRE, and PTPN3 are involved in the regulation of LPS-mediated inflammation [14], [15], [16]. While the mRNA levels of DUSP26 and PTPRE do not change after LPS treatment, PTPN3 mRNA levels increased rapidly after treatment [14], [15], [16]. Nonetheless, overexpression of these PTPs inhibits TNF-α production in RAW 264.7 cells and, therefore, may act as anti-inflammatory regulators.

PTPN7 (also named HePTP, for hematopoietic PTP) is a small, 38 kDa, 339 amino acid, class I non-receptor PTP, which is expressed mainly in the white blood cells of bone marrow, thymus, spleen, lymph nodes, and all myeloid and lymphoid cell lines [17], [18], [19], [20]. PTPN7 mRNA is strongly induced by IL-2 in T-cells with microarray data [21], while the PTPN7 protein regulates IL-2-mediated ERK1/2 signaling because the MAPKs ERK1, ERK2, and p38 are physiological substrates of PTPN7 [20], [22]. Overexpression of PTPN7 in T-cells reduces T-cell receptor (TCR)-induced transcriptional activation by down-regulating ERK1, ERK2 and p38, and negatively regulates T-cell activation and proliferation [12], [20], [23], [24]. PTPN7 binds ERK and p38 via a short, highly conserved motif in the kinase interaction motif localized between residues 15–30 [22].

In this study, we show that PTPN7 expression changes upon stimulation with LPS and that PTPN7 regulates TNF-α production in RAW 264.7 cells.

## Materials and Methods

### Cell Culture and Transfection

Mouse macrophage-like RAW 264.7 cells were maintained at 37°C in Dulbecco’s modified Eagle’s medium (DMEM, Invitrogen, Carlsbad California) supplemented with 10% fetal bovine serum (FBS, Invitrogen) and penicillin/streptomycin in the presence of 5% CO_2_. For transient transfection, 1.4×10^6^ cells were plated in each 60-mm cell culture plate, grown overnight, and transfected with DNA using FuGENE HD transfection reagent (Roche, Basel, Switzerland) according to the manufacturer’s instructions.

### Plasmid Construction

The N-terminal FLAG-tagged PTPN7 plasmid for expression in mammalian cells was constructed by inserting an *Nde*I–*Bam*HI cDNA amplified by polymerase chain reaction into *Nde*I–*Bam*HI site of the pcDNA3.1/Zeo plasmid (Invitrogen) that was modified to produce FLAG fusion. The nucleotide sequences were verified by DNA sequencing.

### Reagents and Antibodies

Anti-phospho-JNK (Thr-183/Tyr-185), anti-phospho-p38 (Thr-180/Tyr-182), anti-ERK1/2, and anti-phospho-ERK1/2 (Thr-202/Tyr-204) antibodies were from Cell Signaling Technology (Danvers, MA). Anti-JNK and anti-p38 antibodies were from Santa Cruz Biotechnology (Santa Cruz, CA). Anti-FLAG M2 antibody and LPS were from Sigma-Aldrich (St. Louis, MO). The rabbit polyclonal anti-PTPN7 (HePTP) and anti-DUSP1 (MKP-1) were obtained from Santa Cruz Biotechnology.

### Enzyme Linked Immunosorbent Assay (ELISA) of TNF-α

TNF-α protein concentrations were determined by standard sandwich ELISA using antibodies and standards obtained from BD Biosciences (San Diego, CA) and used according to manufacturer’s instructions. Assays were performed on neat and diluted samples in duplicate on 96-well plates. Absorbance was measured by a microplate reader at 450 nm and concentrations were determined by comparison to a standard curve. All experiments were repeated at least three times.

### *In vitro* Protein Phosphatase Assays

For *in vitro* phosphatase assays, RAW 264.7 cells were treated with or without LPS as described above and then harvested in PTP lysis buffer (0.5% NP-40, 0.5% Triton X-100, 150 mM NaCl, 20 mM Tris-HCl (pH 8.0), 1 mM EDTA, 1% glycerol, 1 mM PMSF, and 1 µg/ml aprotinin) for 30 min at 4°C. Cleared cell lysates from centrifugation were immunoprecipitated with rabbit anti-PTPN7 or anti-IgG antibodies (Santa Cruz Biotechnology) followed by incubation with protein A/G agarose for 16 h at 4°C using rotation device. After incubation, immunoprecipitated cell lysates were washed three times with PTP lysis buffer and its phosphatase activities were measured using the substrate 3-O-methylfluorescein phosphate (OMFP; Sigma-Aldrich) in a 96-well microtiter plate assay.

### Immunoblotting Analysis

Samples were run in SDS-10% polyacrylamide gels and transferred to nitrocellulose membrane. The membrane was blocked in 5% nonfat skim milk and incubated with an appropriate antibody, followed by incubation with a secondary antibody conjugated to horseradish peroxidase. The immunoreactive bands were visualized using an ECL system (Pierce, Rockford, IL).

### Reverse Transcription-polymerase Chain Reaction (RT-PCR)

Total RNAs were prepared from cells by Trizol (Roche) and reverse transcription was performed using M-MLV (Invitrogen). PCR for mouse PTPs was carried out using the primers listed in Table 1.

**Table 1:** Primer sequences for PTP genes amplified by RT-PCR.

Gene	Synonyms	Accession number	Primer used for PCR analysis
*PTPN7*	HePTP, LCPTP	NM_080588	Forward: 5′-CAGAGACAGCTGCCAACTCCGG-3′Reverse: 5′-CCATGAGCTGGTGGAGGGTCAG-3′
*DUSP1*	MKP-1, 3CH134, PTPN10,HVH1, CL100	XM_003720	Forward: 5′-CCTGTGGAGGACAACCACAAGG-3′Reverse: 5′-GCTGGCCCATGAAGCTGAAG-3′
*DUSP2*	PAC1	XM_049122	Forward: 5′-GTGCCTGGTTCCAGGAGGC-3′Reverse: 5′-CTCAGTGACACACGACCTGGG-3′
*DUSP3*	VHR, T-DSP11	NM_028207	Forward: 5′-GAGGGAGGGCAGGTCCTTCA-3′Reverse: 5′-GAGGGAGGGCAGGTCCTTCA-3′
*DUSP7*	PYST2, B59, MKP-X	NM_153459.1	Forward: 5′-CCTGCCCTACCTCTACCTCGG-3′Reverse: 5′-CACCACACTTCTTGGAGCGG-3′
*DUSP11*	PIR1	NM_028099.3	Forward: 5′-CTCACCACCGGGGAAGCT-3′Reverse: 5′-GACACCTGGATCCTGGGGC-3′
*DUSP12*	HYVH1, GKAP, LMW-DSP4	AF280810	Forward: 5′-CCAATTTGGAGAGCCTGGCC-3′Reverse: 5′-GCTTCTGCATGAGGTAGGCCAC-3′
*DUSP18*	DUSP20, LMW-DSP20	NM_173745.4	Forward: 5′-GCCAGGCAGTGAAATTCAGATG-3′Reverse: 5′-GCCTCTTGGAGATGAAGGTGG-3′
*DUSP26*	VHP	AK009781	Forward: 5′-GCAAGACAGCCTGTAACCATGC-3′Reverse: 5′-GCACCAGGATCTTCCCTCCTG-3′
*PTPRE*	GLEPP1, PTP-U2, PTPROτ	U40280.1	Forward: 5′-CATTGTGATCGATGCCATGATG-3′Reverse: 5′-GTTGCCCGTCCTCATGTTCTC-3′
*PTPN2*	TCPTP, MPTP, PTP-S	NM_008977.1	Forward: 5′-GGCGCTCTGGCACCTTCTC-3′Reverse: 5′-CATCTGCTGCACCTTCTGAGC-3′
*PTPN3*	PTPH1	NM_011207.2	Forward: 5′-CGAGGACGCCAGCCAGTACTAC-3′Reverse: 5′-CTCCTGATCACCAGGGCCAG-3′
*PTPN18*	PTP-HSCF, PTP20, BDP	NM_011206.2	Forward: 5′-CCAGCTACAGTATATGTCCTGGCC-3′Reverse: 5′-CCTGTACTGCTCCTCTGTCTGCAC-3′

### Quantitative Real-time PCR (qRT-PCR)

Total RNAs were isolated from RAW 264.7 cells by Accuzol (Bioneer Corporation, South Korea). cDNA amplification and polymerase chain reaction (PCR) were performed with Omniscript® RT Kit (Qiagen, Hilden, Germany) and iTaq™ Universal SYBR® Green Supermix (*Bio*-Rad, Hercules, CA). The following primer sets were used: PTPN7 mRNA (forward, 5′-CAGAGACAGCTGCCAACTCCGG-3′; reverse, 5′-CCATGAGCTGGTGGAGGGTCAG-3′); GAPDH mRNA (forward, 5′-GCTCTCTGCTCCTCCTGTTC-3′; reverse, 5′-ACGACCAAATCCGTTGACTC-3′). The data obtained from qRT-PCR were quantified using the comparative threshold (2^−[*ΔΔ*Ct]^) method (Ct represents cycle threshold). Relative mRNA expression levels were normalized with GAPDH. The Ct value for each primer pairs was obtained from samples and averaged. Delta Ct value represented the calculated difference between the average Ct for the PTPN7 and the average Ct for GAPDH as the control for total starting RNA quantity. The delta-delta Ct method of calculation was the used to assess fold-change in gene expression relative to GAPDH gene.

### Knock-down of PTPN7

For RNA interference of PTPN7, RAW 264.7 cells grown at 40% confluences were transfected with 1 µg of non-silencing negative control or PTPN7 siRNAs [#1: 5′ - GAU CUA UCU CAG GGA UGA A (dTdT) - 3′, #2: 5′ – CAG GAU AGG CUU CUA AAG U (dTdT) - 3′] (Bioneer Corporation, Daejeon, South Korea) using FuGENE HD transfection reagent (Roche, Basel, Switzerland). After 48 h of transfection, cell lysates were prepared and subjected to immunoblotting analysis with an anti-PTPN7 antibody.

## Results and Discussion

### PTPN7 Expression in Macrophages is Transiently Down-regulated by LPS

**Figure 1:**
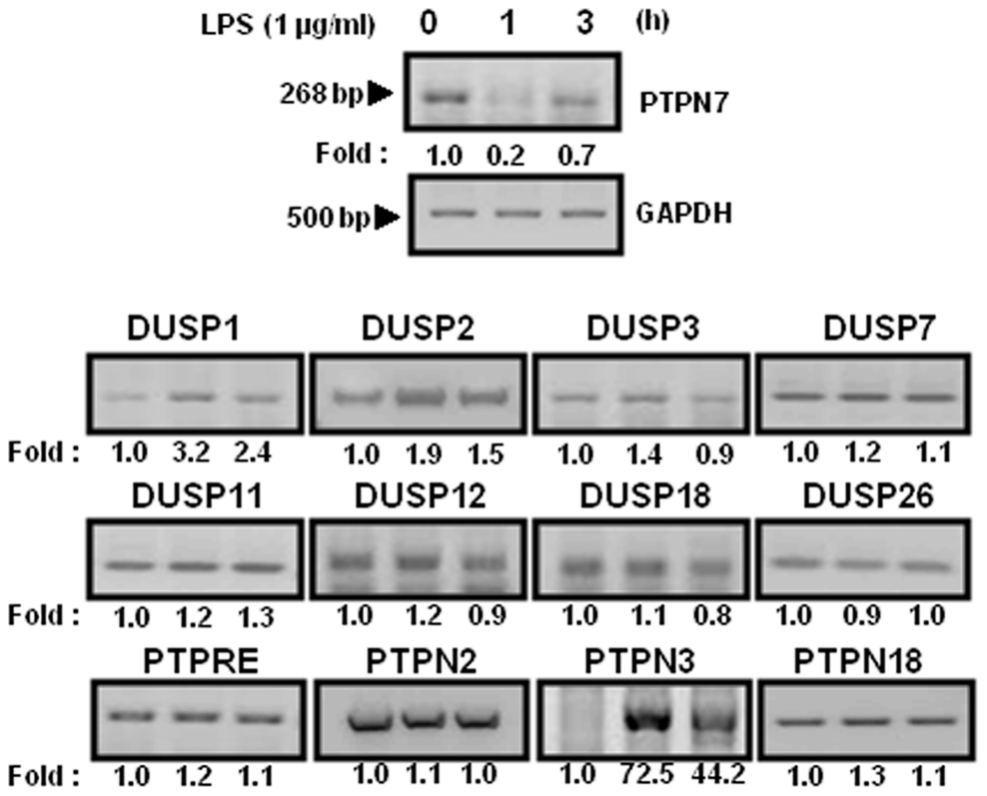
Screening of PTPs regulated in RAW 264.7 cells in response to LPS. Each cDNA was synthesized from total RNA that was extracted from RAW 264.7 cells at the indicated times after treatment with LPS (1 µg/ml). PTP transcripts were amplified using specific primers listed in Table 1. GAPDH transcripts were amplified using specific primers (forward 5′-ACCACCATGGAGAAGGC-3′; reverse 5′-CTCAGTGTAGCCCAGGATGC- 3′) as a control. Similar results were obtained in three independent experiments. The PCR products were visualized by ethidium bromide staining and quantified by scanning the gel images followed by analysis with LabWorks software (UVP Inc.). The PCR data were normalized to the expression level of GAPDH and are presented as relative fold changes.

**Figure 2:**
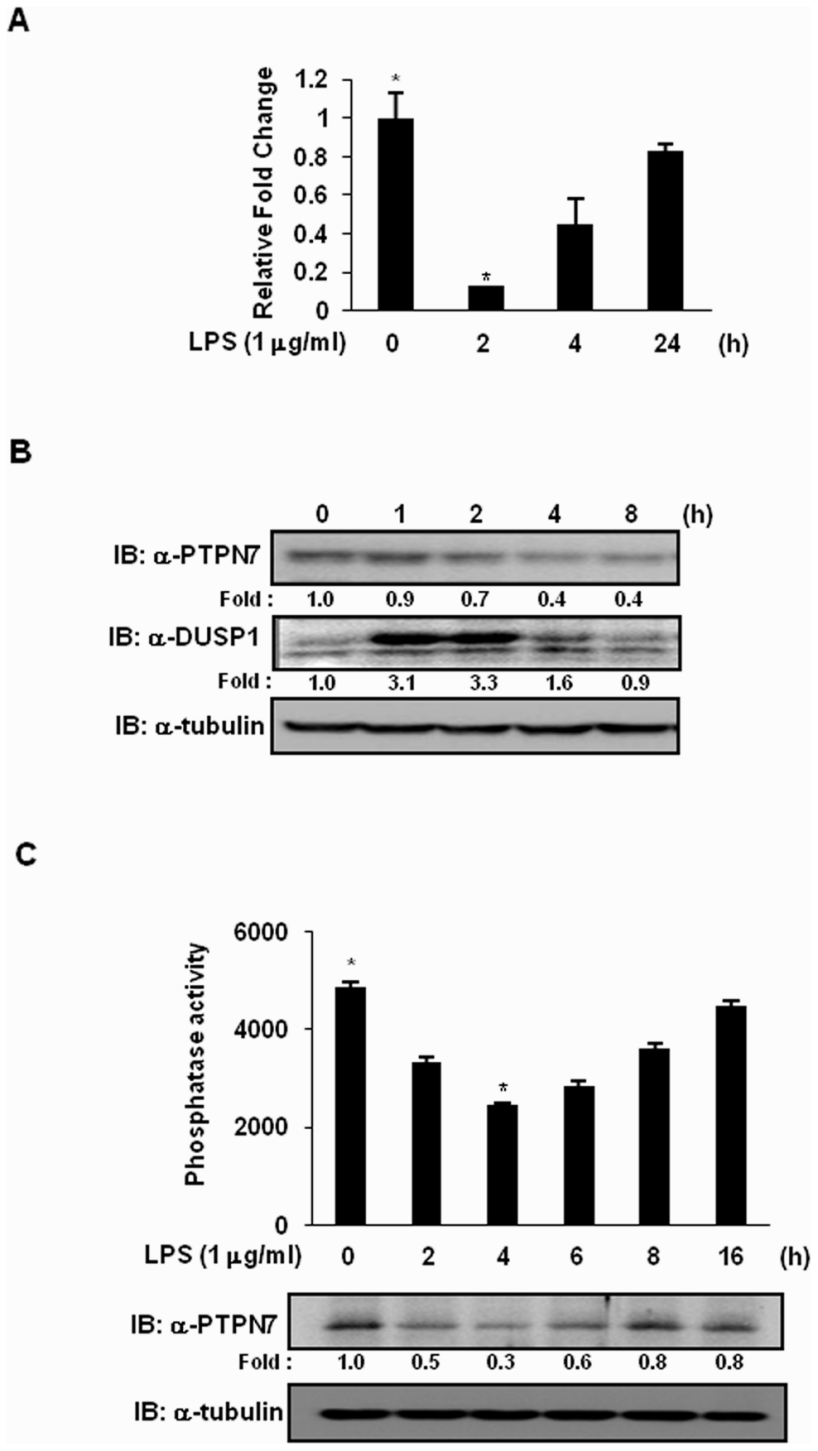
Transient reduction in PTPN7 levels after LPS treatment in RAW 264.7 cells. (A) *PTPN7* transcript level was determined at the indicated times after LPS treatment (1 µg/ml) by quantitative real-time PCR in RAW 264.7 cells. GAPDH transcripts were amplified using specific primers as a control. Relative mRNA expression levels were normalized with GAPDH and presented as fold increase. The results presented represent the mean data from three independent experiments. *p<0.01 versus the untreated sample (Student’s t-test). (B) Expression of PTPN7 in RAW 264.7 cells were analyzed at various time points after LPS stimulation (1 µg/ml) by immunoblotting with appropriate antibodies. The experiments were repeated three times with similar results. Protein expression levels were quantified by scanning the immunoblots followed by analysis with LabWorks software (UVP Inc.). Relative expression levels of PTPN7 and DUSP1 were normalized to the expression level of tubulin and presented as fold increase. IB, immunoblot. (C) After RAW 264.7 cells were untreated or treated with 1 µg/ml of LPS for 2, 4, 6, 8, 16 h, cells were harvested and immunoprecipitated with an anti-PTPN7 antibody. *In vitro* phosphatase activity assays were performed as described in Materials and Methods. The results presented represent the mean data from three independent experiments. *p<0.01 versus the untreated sample (Student’s t-test). Protein expression levels were analyzed by immunoblotting and quantified by scanning the immunoblots followed by analysis with LabWorks software (UVP Inc.).

Signaling pathways in innate immune cells are rapidly activated by phosphorylation, inducing a pro-inflammatory cytokine response to LPS of gram-negative bacteria, and are then down-regulated by the dephosphorylation of signaling components, suggesting that phosphatases are involved in the regulation of inflammation. Therefore, transient regulation of phosphatases during LPS-mediated signaling might be critical for controlling kinase activity. We performed RT-PCR using gene-specific primers against the PTP genes using RNA purified from RAW 264.7 cells, stimulated with LPS for 1 or 3 h (Table 1). Among several PTPs found to be differentially expressed in response to LPS, PTPN7 was one whose expression was regulated upon exposure to LPS (Fig. 1). Interestingly, unlike other regulatory PTPs that were induced by LPS, PTPN7 expression was suppressed (Table 2). PTPN7 expression decreased within 1 h, and then returned to a near basal level by 3 h of LPS stimulation. Using real-time-PCR, we further examined the kinetics of PTPN7 mRNA suppression upon exposure to LPS. Expression of PTPN7 mRNA was clearly suppressed when cells were treated with LPS for 2 h (Fig. 2), but returned to near basal levels when cells were treated with LPS for 24 h (Fig. 2A). When PTPN7 protein expression levels were analyzed by immunoblotting analysis using an anti-PTPN7 antibody, the protein expression pattern was similar to the data obtained from RT-PCR, even though protein expression seemed to be slightly delayed (Fig. 2B). Unlike PTPN7, DUSP1 was induced at early times of LPS treatment and then returned to basal levels after 2 h of LPS treatment. When the endogenous PTPN7 was immunoprecipitated from cells treated with LPS and its phosphatase activities were measured, PTPN7 phosphatase activity decreases in proportion to the decrease of PTPN7 expression (Fig. 2C). Taken together, these results suggest that LPS decreases expression of PTPN7 at an early stage of LPS treatment.

**Table 2:** The list of PTPs analyzed in response to LPS.

Gene	Induction by LPS treatment	References
PTPN7	Red	This study
DUSP1	Ind	This study, [6]
DUSP2	Ind	This study, [10]
DUSP3	No effect	This study, [3]
DUSP4	Ind	[27]
DUSP6	No effect	[3]
DUSP7	No effect	This study
DUSP10	Ind	[28]
DUSP11	No effect	This study
DUSP12	No effect	This study
DUSP14	No effect	[3]
DUSP18	No effect	This study
DUSP22	No effect	[3]
DUSP26	No effect	This study, [14]
PTPRE	No effect	This study, [15]
PTPN2	No effect	This study
PTPN3	Ind	This study, [16]
PTPN18	No effect	This study

Abbreviations: Red, reduced; Ind, induced.

### TNF-α Production is Dependent on PTPN7 Expression Levels

**Figure 3:**
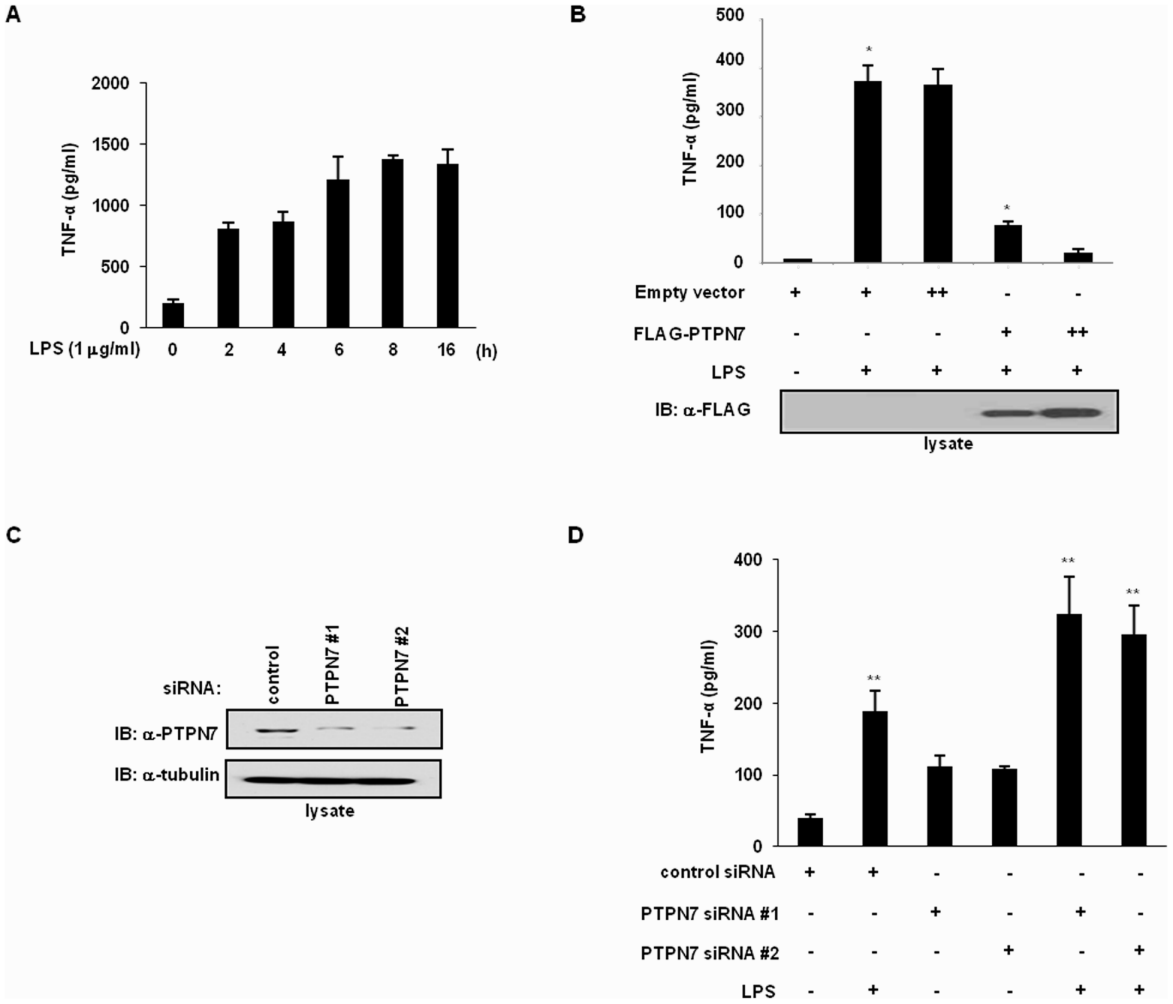
Inhibition of LPS-stimulated TNF-α production by PTPN7 in RAW 264.7 cells. (A) Secretion levels of LPS-stimulated TNF-α were increased time-dependently in cell culture supernatants. At different times post treatment with LPS, culture supernatants were analyzed for TNF-α production using ELISA assay. (B) RAW 264.7 cells were transfected with either the empty vector or the construct expressing FLAG-PTPN7. After 16 h stimulation with LPS (1 µg/ml), supernatants were analyzed for TNF-α production using an ELISA assay, as described under Materials and Methods. Cell lysates were subjected to immunoblotting using an anti-FLAG antibody for detection of PTPN7. The results presented represent the mean data from three independent experiments. *p<0.01 versus the vector controls (Student’s t-test). (C) PTPN7 knockdown (PTPN7 siRNA #1 and #2) was detected via immunoblotting using anti-PTPN7 and anti-tubulin antibodies. (D) After transfection with control or PTPN7 siRNAs (#1 and #2), cells were treated with LPS (1 µg/ml) for 1 h, and the levels of TNF-α were measured using an ELISA assay. The results presented represent the mean data from three independent experiments. **p<0.05 versus the LPS-treated control siRNA (Student’s t-test).

TNF-α production is one of the earliest pro-inflammatory responses from macrophages when stimulated by endotoxins such as LPS. Since LPS regulates PTPN7 expression in RAW 264.7 cells, we hypothesized that PTPN7 might be involved in the regulation of TNF-α expression. To investigate the effect of PTPN7 on TNF-α expression, we measured the time point changes of TNF-α and PTPN7 in response to LPS (Fig. 3A). TNF-α levels increased sharply at early times of LPS treatment, then increased gradually and reached maximal point at 8 h after LPS treatment. The increase in TNF-α levels after 4 h of treatment coincides with the decrease in PTPN7 levels (Fig. 2B). To assess the effects of PTPN7 expression on TNF-α production, we analyzed the levels of TNF-α in RAW 264.7 cells transfected with a plasmid which coded for PTPN7 expression. RAW 264.7 cells transfected with a mammalian vector expressing FLAG-tagged PTPN7 were treated with 1 µg/ml LPS, and the effect of increased PTPN7 expression on TNF-α secretion in the medium was examined by ELISA. Compared to cells transfected with an empty plasmid, cells expressing FLAG-PTPN7 exhibited a 90% reduction in TNF-α production in response to LPS stimulation (Fig. 3B). To confirm that PTPN7 regulates TNF-α production, PTPN7-specific small interfering RNAs (siRNAs) were transfected into RAW 264.7 cells to knock-down PTPN7. Reduced levels of PTPN7 expression were confirmed by immunoblotting analysis (Fig. 3C). Following transfection with either non-targeting control siRNA or PTPN7-specific siRNAs (#1 and #2), RAW 264.7 cells were treated with LPS, and TNF-α levels in the medium were measured (Fig. 3D). As shown in Fig. 3D, knock-down of PTPN7 enhanced LPS-induced TNF-α production in RAW 264.7 cells. Taken together, these results suggest that PTPN7 inhibits LPS-stimulated TNF-α induction in macrophages.

### PTPN7 Regulates MAPK Signal Transduction

**Figure 4:**
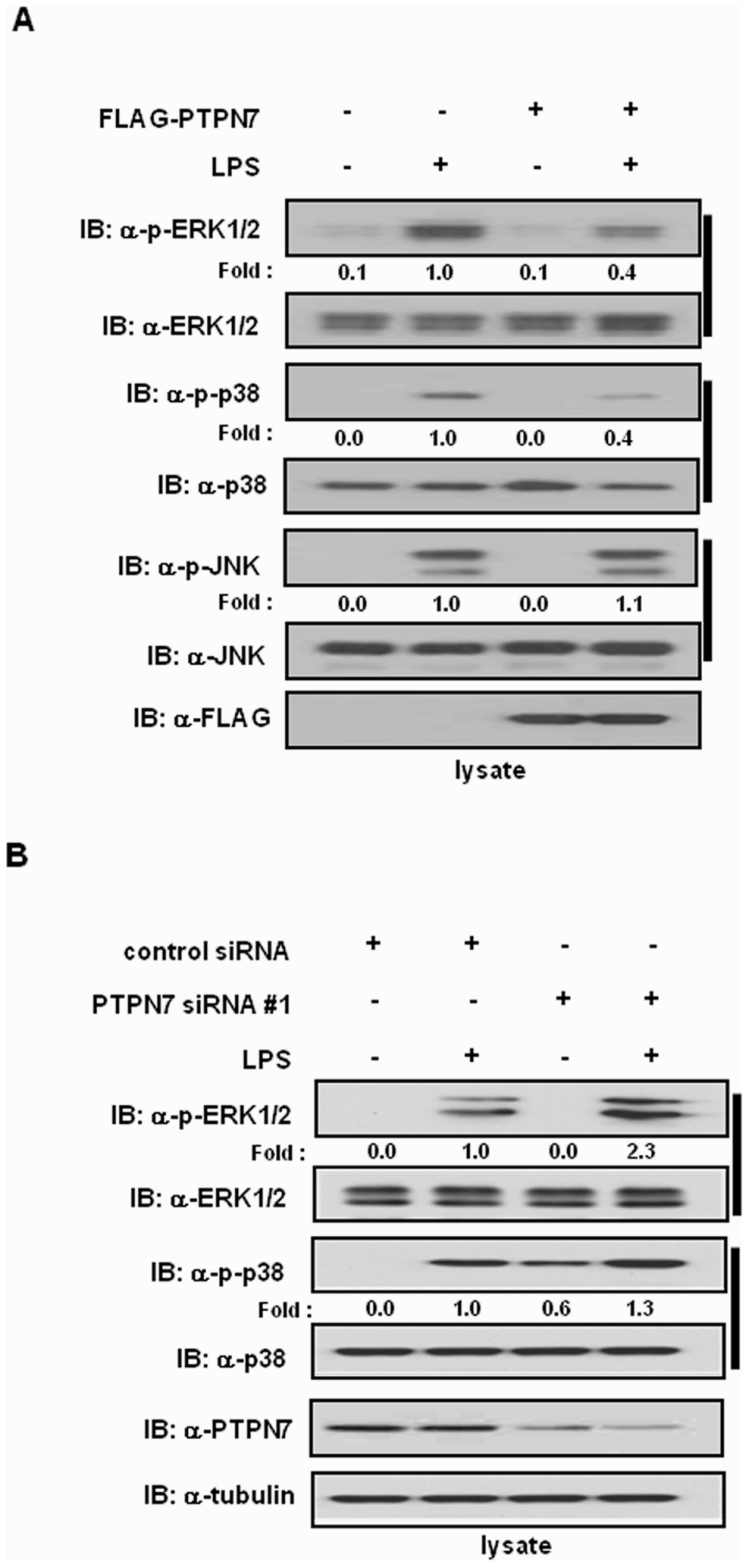
Effect of PTPN7 on LPS-mediated MAPK signal transduction. (A) After PTPN7-transfected RAW 264.7 cells were stimulated with LPS (1 µg/ml) for 1 h, immunoblotting was performed for total and phosphorylated proteins as indicated. Relative phosphorylation levels of MAPKs were normalized to the expression levels of the corresponding total MAPKs and presented as fold increase. Data are representative of four independent experiments. (B) RAW 264.7 cells were transfected with control siRNA or PTPN7 siRNA #1 for 48 h, and were stimulated with LPS (1 µg/ml) for 1 h. MAPK activities were determined by immunoblotting with appropriate antibodies. Relative phosphorylation levels of MAPKs were normalized to the expression levels of the corresponding total MAPKs and presented as fold increase. Data are representative of four independent experiments.

Since PTPN7 has been known to selectively dephosphorylate a phospho-tyrosine residue on the activation loop of the MAPKs ERK1/2 and p38 [25], [26], we examined the effect of PTPN7 on MAPK signaling in LPS-stimulated RAW 264.7 cells. RAW 264.7 cells transfected with either FLAG-tagged PTPN7 or empty plasmids were stimulated with LPS, and endogenous levels of phospho-JNK, phospho-p38, and phospho-ERK1/2 were assessed by immunoblotting with phospho-ERK1/2, phospho-JNK, and phospho-p38 antibodies. Compared to cells transfected with empty plasmid, the levels of phospho-ERK1/2 and phospho-p38 were reduced in PTPN7-transfected cells, whereas levels of phosphorylated JNK were not changed (Fig. 4A). In contrast, knock-down of PTPN7 enhanced levels of phopho-ERK1/2 and phopho-p38 in LPS–stimulated cells (Fig. 4B). Taken together, these results indicate that the activities of ERK1/2 and p38 were down-regulated by PTPN7 in LPS-stimulated macrophages. Therefore PTPN7 acts as a negative regulator of MAPKs, and particularly of ERK1/2 and p38 in macrophages.

The expression of PTPN7 was dramatically diminished soon after LPS stimulation, but gradually recovered to normal levels. Down-regulation of PTPN7 leads to the production of TNF-α, and an increase in MAPK phosphorylation in LPS-treated cells. However, unlike PTPN7, expression of other phosphatases, such as DUSP1, was enhanced in response to LPS stimulation. DUSP1, which was induced within 1 to 2 h of LPS treatment, down-regulated JNK and p38 activities and TNF-α production in RAW 264.7 cells [11]. Therefore, both PTPN7 and DUSP1 inactivate MAPK signaling, even though there is a slight difference in substrate specificities. However, the expression patterns of these phosphatases in response to LPS are opposite: PTPN7 is suppressed after 2 h of LPS treatment while DUSP1 is induced at early times of LPS treatment. Why cells express conflicting responses to LPS is not yet clear. One possible explanation may be that PTPN7 has a role as an intrinsic inhibitor of MAPK signaling, and its down-regulation activates MAPK for TNF-α production. It is also possible that the kinetics of *PTPN7* gene suppression might be different from that of *DUSP1* gene induction in response to LPS as shown in Fig. 2B. Differential and time-dependent expression of these PTPs might be important to regulate inflammation. Another possibility is that other substrates for PTPN7 besides ERK and p38 might be involved in PTPN7-mediated inflammatory responses.

In summary, the present study shows that one important function of PTPN7 is the regulation of the inflammatory response in mouse macrophage cells. PTPN7 expression was potently reduced at early time points after LPS stimulation in RAW 264.7 cells, and is an important regulator of TNF-α production. PTPN7 also functions as a negative regulator of MAPK activity, especially of ERK1/2 and p38. Based on these data, we suggest that PTPN7 is one of key regulators of TNF-α expression and MAPK signal transduction in the inflammatory response of macrophages.
